# Most microRNAs in the single-cell alga *Chlamydomonas reinhardtii* are produced by Dicer-like 3-mediated cleavage of introns and untranslated regions of coding RNAs

**DOI:** 10.1101/gr.199703.115

**Published:** 2016-04

**Authors:** Adrian A. Valli, Bruno A.C.M. Santos, Silvia Hnatova, Andrew R. Bassett, Attila Molnar, Betty Y. Chung, David C. Baulcombe

**Affiliations:** 1 Department of Plant Sciences, University of Cambridge CB2 3EA, Cambridge CB2 3EA, United Kingdom

## Abstract

We describe here a forward genetic screen to investigate the biogenesis, mode of action, and biological function of miRNA-mediated RNA silencing in the model algal species, *Chlamydomonas reinhardtii*. Among the mutants from this screen, there were three at *Dicer-like 3* that failed to produce both miRNAs and siRNAs and others affecting diverse post-biogenesis stages of miRNA-mediated silencing. The DCL3-dependent siRNAs fell into several classes including transposon- and repeat-derived siRNAs as in higher plants. The DCL3-dependent miRNAs differ from those of higher plants, however, in that many of them are derived from mRNAs or from the introns of pre-mRNAs. Transcriptome analysis of the wild-type and *dcl3* mutant strains revealed a further difference from higher plants in that the sRNAs are rarely negative switches of mRNA accumulation. The few transcripts that were more abundant in *dcl3* mutant strains than in wild-type cells were not due to sRNA-targeted RNA degradation but to direct DCL3 cleavage of miRNA and siRNA precursor structures embedded in the untranslated (and translated) regions of the mRNAs. Our analysis reveals that the miRNA-mediated RNA silencing in *C. reinhardtii* differs from that of higher plants and informs about the evolution and function of this pathway in eukaryotes.

RNA silencing in eukaryotes controls gene expression and protects against viruses and transposons (Baulcombe 2004). Small (s)RNAs of 20–31 nucleotides (nt) form RNA-induced silencing complexes (RISC) with proteins of the Piwi/Argonaute family (AGO) and they guide these effector proteins to their targets by complementary base-pairing (Meister 2013). AGO proteins achieve posttranscriptional gene silencing (PTGS) by target transcript degradation or translational repression and they promote transcriptional gene silencing (TGS) via chromatin/DNA modifications (Brodersen and Voinnet 2009; Castel and Martienssen 2013).

Corresponding to these various RNA silencing pathways, there are multiple types of sRNA that differ in their biogenesis mechanism or in their associated AGO isoform. These sRNAs include small interfering (si)RNAs, micro(mi)RNAs, and piwi-interacting (pi)RNAs (Ghildiyal and Zamore 2009). The siRNAs and miRNAs are produced by the action of RNase III Dicer (Dcr) or Dicer-like (DCL) proteins on fully- or near-complementary double-stranded (ds)RNA molecules (Carthew and Sontheimer 2009), whereas piRNAs are Dcr-independent and have single-stranded RNA precursors (Iwasaki et al. 2015).

The miRNAs of plants and animals are similar: They are 20–24 nt and derived from precursor RNAs with stem–loop structures (Brodersen and Voinnet 2009). However, there are also clear differences. The biogenesis of animal miRNAs, for example, involves processing of a primary miRNA transcript by various nucleases, including the microprocessor Drosha/DGCR8 to form a miRNA precursor that is then cleaved by Dcr in the cytoplasm (Bartel 2009). In contrast, plant miRNAs are processed in a nuclear DCL-mediated mechanism (Brodersen and Voinnet 2009). There are other differences based on the composition of the AGO complex, requirement for sequence complementarity between the miRNA and its target, and the ways that translation is suppressed. These differences prompted the speculation that miRNAs have evolved independently in plant and animal lineages (Axtell et al. 2011).

Most information about miRNAs is from multicellular organisms, although they are also present in unicellular organisms, including the green alga *Chlamydomonas reinhardtii* (Molnár et al. 2007; Zhao et al. 2007), protozoans *Giardia lamblia* (Saraiya and Wang 2008), *Trichomonas vaginalis* (Chen et al. 2009), *Pentatrichomonas hominis* (Huang et al. 2012), *Symbiodinium microadriaticum* (Baumgarten et al. 2013), *Entamoeba histolytica* (Mar-Aguilar et al. 2013), *Trypanosoma brucei* (Mallick et al. 2008), and *Toxoplasma gondii* (Braun et al. 2010). These organisms are descended from ancient ancestors of multicellular organisms, and they provide an opportunity to test hypotheses about the origin of miRNA pathways.

Here we focus on *C. reinhardtii*, which is from a lineage that diverged from the ancestor of land plants more than one billion years ago (Yoon et al. 2004). It has a complex RNA silencing machinery with three DCLs (DCL1-3) and three AGOs (AGO1-3) (Merchant et al. 2007; Casas-Mollano et al. 2008). These proteins are not encoded by orthologs of genes in higher plants, although it is well established that *C. reinhardtii* sRNAs, including miRNAs, are like those of higher plants in that they direct cleavage of their mRNA targets (Molnár et al. 2007; Zhao et al. 2007). To investigate the biogenesis, mode of action, and biological function of miRNAs in *C. reinhardtii* we have carried out a forward genetic screen in this genetically tractable organism.

## Results

### Isolation of RNA silencing mutants in *C. reinhardtii*

**Figure 1.**
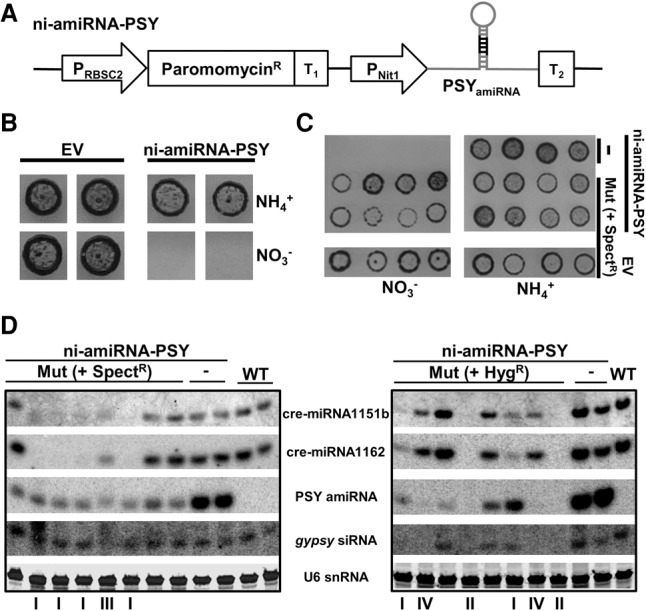
Screening and isolation of mutants affected in miRNA-mediated RNA silencing. (*A*) Schematic representation of the artificial miRNA construct used to transform the wild-type strain. Transgenic lines carrying this cassette were further screened by random insertional mutagenesis: (P_RBSC2_) RuBisCO small subunit (RBSC)2 promoter; (Paromomycin^R^) *Streptomyces rimosus* AphVIII coding gene; (T_1_) RBSC2 transcription terminator; (P_Nit1_) nitrate reductase promoter; (PSY_amiRNA_) modified version of cre-miR1157 that carries a miRNA against the phytoene synthase; (T_2_) RLP12 transcription terminator. (*B*) Selective cell death of transgenic lines expressing the PSY amiRNA in the presence of nitrate, but not ammonium, as the sole nitrogen source. Transgenic lines carrying the empty amiRNA vector (EV) were used as control. (*C*) Growth in high light conditions of mutagenized (Spect^R^) and nonmutagenized (-) reporter lines (PSY_amiRNA_) in solid media containing either nitrate or ammonium as sole nitrogen source. Transgenic lines carrying the empty amiRNA vector (EV) and further transformed with the spectinomycin resistance cassette were used as control. (*D*) Detection by Northern blot of diverse small RNAs in total RNA samples from the indicated mutants and controls. These mutants were obtained by random insertional mutagenesis of either spectinomycin or hygromycin resistance cassettes. The mutants were grouped (I–IV) (Supplemental Table S1) based on the molecular phenotype. The two displayed mutants belonging to the group II correspond to the characterized mutant 47 (*dcl3-2*) and mutant 51 (*dcl3-3*).

To characterize mechanisms and biological function of RNA silencing in *C. reinhardtii* we used a reporter system in which a nitrate-inducible artificial (a)miRNA was targeted to the 5′ region of the phytoene synthase (*PSY*) mRNA (ni-amiRNA-PSY) (Fig. 1A; Supplemental Fig. S1). The amiRNA was readily detectable by Northern blotting in cells using nitrate rather than ammonium as a source of nitrogen and, correspondingly, from qRT-PCR the *PSY* mRNA was less abundant in nitrate-grown cells (Supplemental Fig. S1A,C). From these data we conclude that the amiRNA down-regulated the *PSY* mRNA. We confirmed this conclusion by 5′-RACE detection of *PSY* mRNA cleavage products at the amiRNA target site (Supplemental Fig. S1D).

The amiRNA-producing cells died in light in the presence of nitrate (Fig. 1B) most likely due to silencing of *PSY* mRNA by the amiRNA and to the consequent lack of the photoprotective function of PSY (McCarthy et al. 2004). Consistent with this interpretation the cell death was dependent on the light intensity (Supplemental Fig. S1B), and it did not occur in cells using ammonium rather than nitrate as nitrogen source where the amiRNA promoter is repressed (Fig. 1B). We therefore used the light-induced cell death to screen for mutants in amiRNA silencing pathways.

Two independent amiRNA lines (named A4-1 and E9-3) were mutagenized by random genomic insertion of either spectinomycin or hygromycin resistance cassettes. The mutagenized cells grew well on solid medium with ammonium as nitrogen source but, unlike cultures of wild-type cells expressing the amiRNA, there were some cells that grew in nitrate (Fig. 1C). We hypothesized that amiRNA silencing of *PSY* had failed in these nitrate-tolerant cells due to a mutation either in the amiRNA gene, in the amiRNA biogenesis pathways, or in the effector machinery of amiRNA silencing.

To further characterize 48 of these nitrate-tolerant lines, we used Northern blotting with probes for the *PSY* amiRNA, cre-miR1151b, cre-miR1162, and for a siRNA from a *gypsy* transposon locus (Fig. 1D). Of these lines, 22 were depleted in the *PSY* amiRNA but without any effect on the endogenous sRNAs. These mutants are likely to affect the amiRNA gene and were not analyzed further. In the other lines, the amiRNA and endogenous sRNAs were reduced to different extents: Group I mutants had reduced levels of miRNAs but not the siRNA; group II were depleted for all tested sRNAs; group III sRNAs were slightly less abundant than in wild-type cells and they were heterodispersed in size; and group IV sRNAs were depleted for the *gypsy* siRNA and amiRNA and had reduced levels of endogenous miRNAs (Fig. 1D; Supplemental Table S1). From these data, we conclude that there may be separate but overlapping pathways for miRNA- and siRNA-mediated silencing. The mutant cells grew well, and we further conclude that these RNA silencing pathways are not required for normal growth of the algal cells in solid or liquid media in normal laboratory conditions.

### Mapping of DCL3 mutants

**Figure 2.**
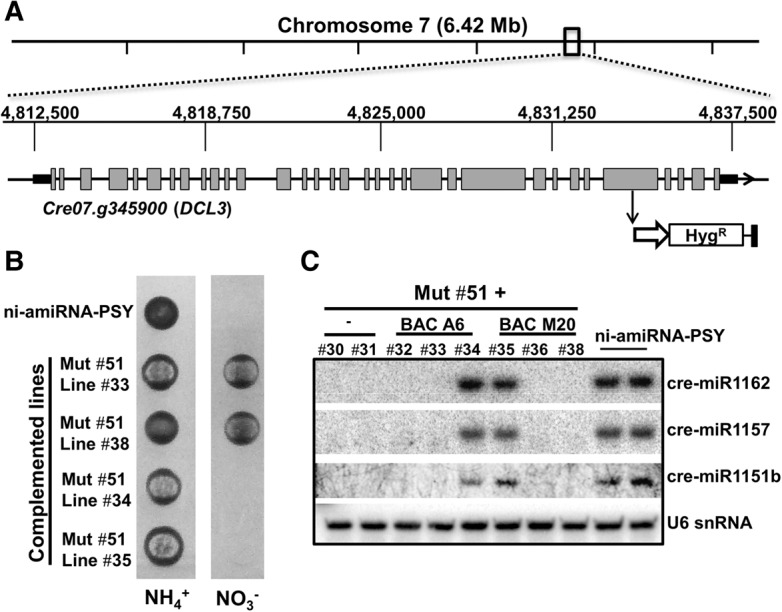
Mapping and complementation of group II mutant 51. (*A*) Location of the mutagenic hygromycin resistance cassette in mutant 51. (*B*) Phenotype of the indicated parental line and both complemented and noncomplemented lines (biological triplicates) in the presence of either nitrate or ammonium under high light conditions. (*C*) Detection by Northern blot of the indicated miRNAs in total RNA samples from the *C. reinhardtii* strains analyzed in *B*.

Because mutant strains in group II displayed the most severe molecular phenotype, we decided to characterize them in detail. Restriction enzyme site-directed amplification (RESDA-)PCR revealed that three group II mutations were in *DCL3*. The mutagenic inserts were in exon 29 (mutant 51) (Figs. 1D, 2A); the 3′ UTR with a deletion that extended to the 5′ end of its neighbour gene *Cre07.g345900* (mutant 47) (Fig. 1D); and exon 6 (mutant 37) (Supplemental Fig. S2A). The *PSY* mRNA was at wild-type levels in these lines (Supplemental Fig. S2B) and, corresponding to the absence of the amiRNA, we could not detect the miRNA cleavage products of the *PSY* mRNA (Supplemental Fig. S2C).

Final confirmation of *DCL3* mutation was by complementation of mutant 51 with bacterial artificial chromosomes (BACs) (BAC A6 and BAC M20) carrying the genomic sequence corresponding to *DCL3* (*Cre07.g345900*). After transformation of mutant 51 only two independent colonies had the extra copy of *DCL3* in their genome. Importantly, these complemented lines were light sensitive when PSY amiRNA was induced with nitrate (Fig. 2B), and they regained the capacity to produce endogenous miRNAs (Fig. 2C). Henceforth, we refer to the original lines isolated from the screen as carrying *dcl3-1* (mutant 37), *dcl3-2* (mutant 47), and *dcl3-3* (mutant 51).

*C. reinhardtii* DCL3 has the typical DCL domain organization except that, like the other DCLs in this alga, it lacks a PAZ domain that could be detected by primary and secondary structure prediction algorithms (Supplemental Fig. S3A). This protein is also exceptional among other DCL proteins in that it has a proline rich region (39/52 residues) on the amino terminal side of the RNase III motifs although a similar domain is also found in a related protein, Drosha. Drosha also has RNAse III motifs and it is involved in the first steps of the animal miRNA biogenesis pathway (Supplemental Fig. S3A,B).

### DCL3 and sRNA biogenesis

**Figure 3.**
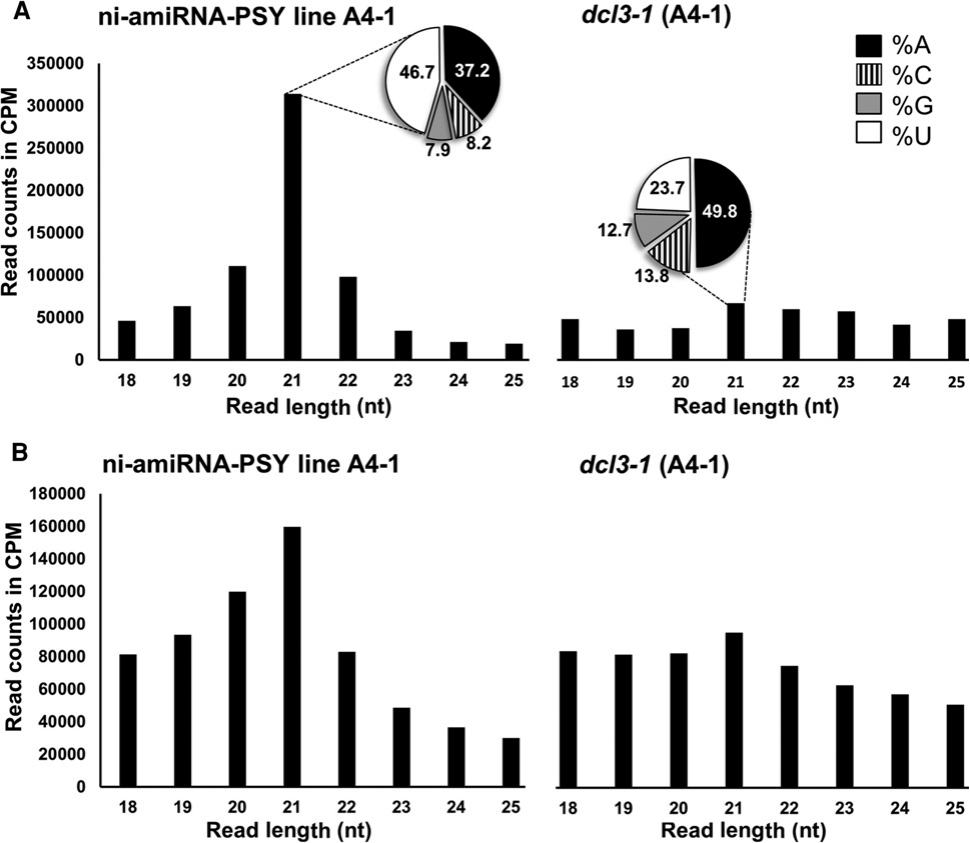
Effect of *dcl3* mutation on *C. reinhardtii* small RNA population. (*A*) Size-distribution histograms of sRNAs from the parental line A4-1 and its derivative *dcl3-1* mutant expressed as the number of counted reads of a given size per million (CPM) of reads matching the *C. reinhardtii* genome. The percentage of 21-nt sRNAs with their 5′ nucleotide identities is also shown. (*B*) Size-distribution histograms of nonredundant sRNAs from the parental line A4-1 and its derivative *dcl3-1* mutant expressed as CPM of reads matching the *C. reinhardtii* genome. Two additional replicates per sample, as well as three replicates from the E9-3 parental and *dcl3-3* lines, showed the same result.

The Northern blot analysis indicated a requirement of DCL3 for biogenesis of both siRNAs and miRNAs (Fig. 1D). To extend this analysis on a genome-wide basis, we sequenced sRNAs from two wild-type parental lines and two *dcl3* lines (*dcl3-1* and *dcl3-3*). Consistent with previous reports (Molnár et al. 2007; Zhao et al. 2007), the sRNAs from lines expressing the amiRNA were mostly 20–22 nt long with a clear peak at 21 nt that was absent in the *dcl3* mutants. As observed previously, the 21-nt sRNAs had a bias toward U or A as first nucleotide (Molnár et al. 2007; Zhao et al. 2007) and those with a 5′ U were clearly reduced in *dcl3* mutants (Fig. 3A). The heterogeneity of both 20- and 21-nt-long small RNAs was also diminished in *dcl3* mutants, as observed in the analysis of nonredundant reads (Fig. 3B).

To identify the DCL3-dependent sRNA loci we aligned libraries of sRNA sequence from wild-type and *dcl3* lines to the reference genome of *C. reinhardtii*. There were 5152 sRNA loci identified in all samples, of which 4313 (83.7%) were differentially expressed between the wild-type parental cells and the *dcl3* mutant lines. The majority of these, 3366 (65.3%), were expressed at a higher level in the parental lines than in the mutant.

To evaluate the effect of *dcl3* loss of function on miRNA production, taking into account a controversy about the number of miRNA genes in *C. reinhardtii* (Nozawa et al. 2012; Taylor et al. 2014), we carried out a stringent de novo prediction of miRNAs from all the identified sRNA loci present in both wild-type and mutant-derived samples (see Methods). This prediction indicated the presence of 18 canonical miRNA loci in *C. reinhardtii*, named in this paper as “high confidence miRNAs” (Table 1). These high confidence miRNAs include seven of the nine miRNAs identified by Taylor et al. (2014), as well as other previously reported/predicted miRNAs. Northern blot confirmed the production, as well as DCL3-dependency, of three of four novel high-confidence miRNAs found by our prediction tool (Table 1; Supplemental Fig. S4). Twenty-four additional loci specified precursor RNAs with miRNA-like features, but lacking a miRNA*, with more than one major sRNA species per arm, or with a variable 5′ end. These candidate miRNA loci were assigned to “medium confidence miRNAs” (Table 1). Only 16 of the 50 miRNA precursors currently annotated in miRBase v.21 were identified by our stringent prediction and, in agreement with a previous analysis (Taylor et al. 2014), it is likely that the others are misannotated siRNA loci.

**Table 1.**
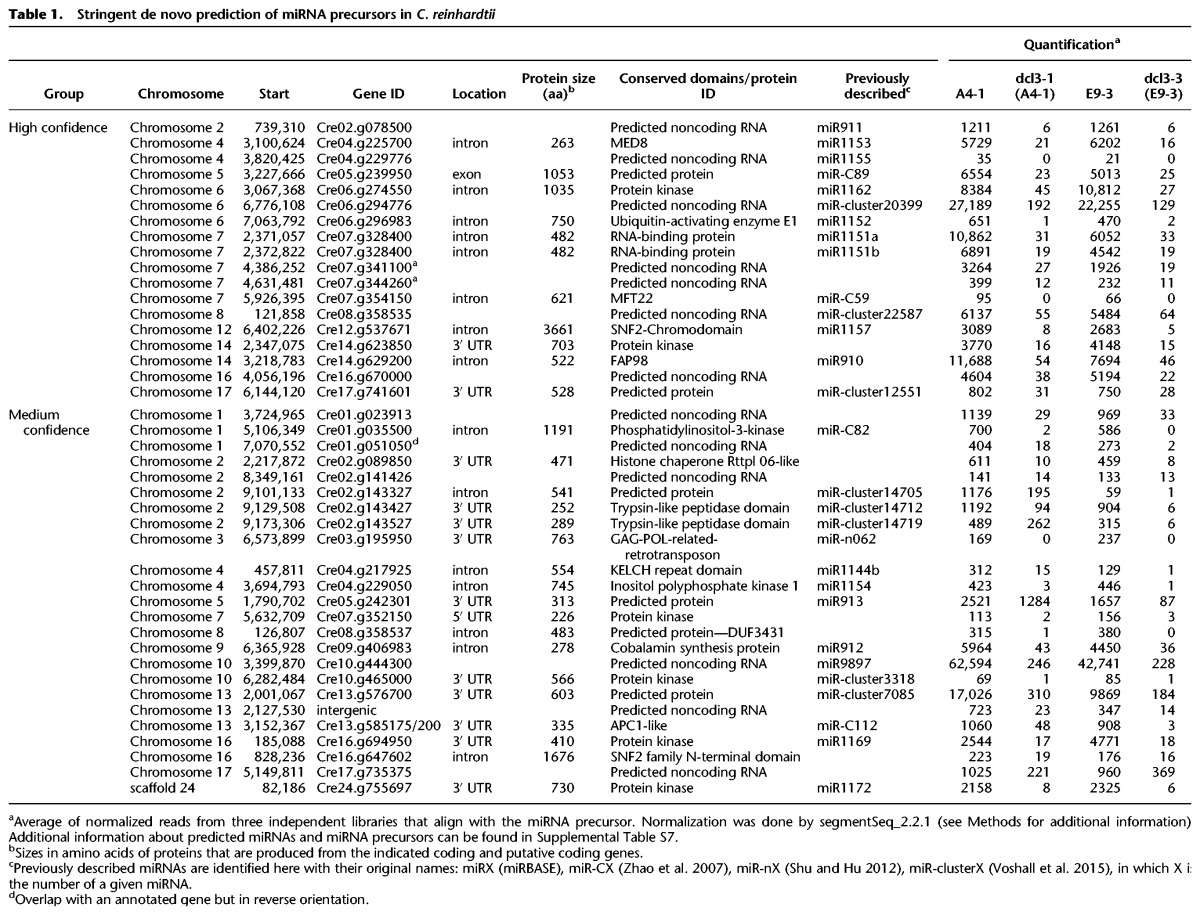
Stringent de novo prediction of miRNA precursors in *C. reinhardtii*

The miRNAs or candidate miRNAs from all class loci were less abundant in *dcl3-1* and *dcl3-3* cells than in the corresponding parental lines (Table 1; Supplemental Table S2). Many (61.1%) of the high confidence miRNAs were derived from introns (nine miRNAs) or UTRs (two miRNAs) of mRNA precursors. The medium confidence miRNA candidates were also from mRNA precursors (75%) but they corresponded to UTRs (11 miRNAs) more than introns (seven miRNAs). The remaining miRNAs in both classes fell into a more canonical class derived from noncoding RNAs (Table 1).

We refer to the non-miRNAs as siRNAs and we classified the genomic siRNA loci into three major classes corresponding to protein-coding genes, transposable elements, and repeat elements. We further classified transposons and repeat associated siRNAs based on the output of RepeatMasker (Supplemental Table S2). All types of siRNA were predominantly dependent on DCL3, including *gypsy* siRNAs (Fig. 1). However, there were some protein-coding genes and non-LTR transposons (SINEX, RE, RTE) at which siRNA production was as great or greater in the *dcl3* mutants than in the wild-type parents (DE dcl3>wt and NDE in Supplemental Table S2). These DCL3-independent siRNAs, as well as the marginal amount of miRNAs produced in *dcl3-1* and *dcl3-3* (Table 1), were presumably generated either by DCL1 or DCL2.

### Processing of intron-derived miRNAs in *Chlamydomonas*

**Figure 4.**
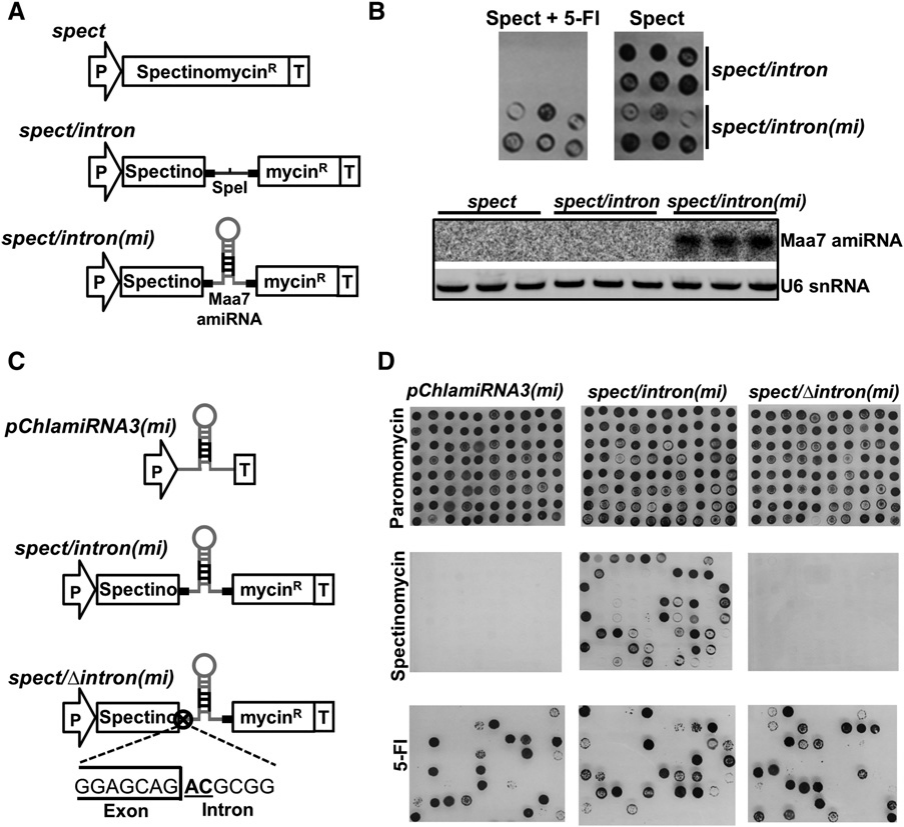
The cre-miR1157 is an intron-derived miRNA. (*A*) Schematic representation of constructs carrying the cre-miR1157 intron inserted into the spectinomycin resistance gene coding sequence. The cre-miR1157 intron was modified to either lack the miRNA stem–loop or carry an artificial miRNA against *Maa7* in *spect/intron* and *spect/intron(mi)* plasmids, respectively: (P) Hybrid RBSC2/HSP70A promoter; (Spectinomycin^R^) recoded *Escherichia coli*-derived *aadA* coding gene; (T) RBSC2 transcription terminator; (SpeI) unique cleavage site for *Spe*I restriction enzyme; (*Maa7* amiRNA) modified version of cre-miR1157 that carries a miRNA against *Maa7*. (*B*, *top*) Growth of the indicated transgenic lines in solid media carrying spectinomycin with/without 5-Fluorindole (5-Fl). (*Bottom*) Detection by Northern blot of the artificial miRNA against *Maa7* in total RNA samples from the indicated lines (three independent lines per construct). (*C*) Schematic representation of constructs used to test the requirement of splicing for the expression of id-miRNA. The GT × AT point mutations in the exon/intron junction are indicated. These plasmids also carry the Paromomycin^R^ cassette (equivalent to the cassette showed in Fig. 1A) to allow the primary selection of transgenic lines in paromomycin. (*D*) Growth of lines transformed with the indicated plasmids in solid media containing either paromomycin (test for plasmid integration), spectinomycin (test for splicing events), or 5-Fl (test for amiRNA production).

Intron-derived (id-)miRNAs are not unique to *C. reinhardtii*, they are also found in animals. The maturation of id-miRNAs in animals referred to as miRtrons (Ruby et al. 2007) is linked to intron splicing. To investigate this possibility in *C. reinhardtii*, we assembled a spectinomycin resistance gene with a miRNA-containing intron embedded in the coding sequence (*spect/intron(mi)*). The intron was from a *C. reinhardtii* gene (*Cre12.g537671*) and it contained the stem–loop RNA that is the precursor of the high confidence cre-miR1157 but with the miRNA sequence modified to target the mRNA of the tryptophan synthase beta-subunit (Maa7) (Fig. 4A). Silencing of *Maa7* confers resistance to 5-fluoro indole (5-FI) (Rohr et al. 2004). Control constructs either lacked an intron (*spect*) or had an intron without the miRNA stem–loop (*spect/intron*) (Fig. 4A).

The id-miRNA was spliced efficiently from these RNAs because the *spec/intron(mi)* construct conferred spectinomycin resistance as efficiently as the *spec* and *spec/intron* controls (Supplemental Fig. S5A). RT-PCR further confirmed correct splicing of the id-miRNA (Supplemental Fig. S5B,C), and a sRNA Northern blot (Fig. 4B) showed, as predicted, production of the mature Maa7 amiRNA. The id-miRNA was fully functional as it silenced the *Maa7* mRNA so that the *spec/intron(mi)* cells were resistant to 5-Fl. Cells with the control constructs without the id-miRNA did not produce the amiRNA, and they were fully susceptible to 5-FI (Fig. 4B).

Finally, to analyze the requirement for splicing in miRNA biogenesis, we generated an id-miRNA construct with a mutation in the splice donor site (Fig. 4C, *spec/Δintron(mi)*). This construct conferred resistance to 5-FI but, as expected, not to spectinomycin (Fig. 4D). From our results in Figure 4 and Supplemental Figure S5, it is clear that the presence of the id-miRNA does not prevent the intron processing and that, unlike animal miRtrons, the intron processing is not required for miRNA biogenesis.

### Differential gene expression in *dcl3* mutants

To identify mRNA targets of miRNAs, we used RNA-seq of the transcriptome in *dcl3* mutant and parental lines. There were 118 annotated genes with statistically significant differences (equal to or greater than 0.9 likelihood) in abundance between the *dcl3-1* and *dcl3-3* mutants and the corresponding wild-type parental cells (Supplemental Table S3).

The 118 DCL3-sensitive RNAs were in several classes corresponding to the following:

1. Noncoding RNAs with miRNA precursors (five genes);
2. Noncoding RNAs with siRNA precursors (64 genes);
3. mRNAs with miRNA precursors in the exons corresponding to the coding sequence (one gene) and 3′ UTR (eight genes);
4. mRNAs with siRNA precursors in the exons corresponding to the 5′ UTR (five genes), coding sequence (three genes), and 3′ UTR (21 genes); and
5. mRNAs with fold back RNA structures producing no clear siRNAs (nine genes).

**Figure 5.**
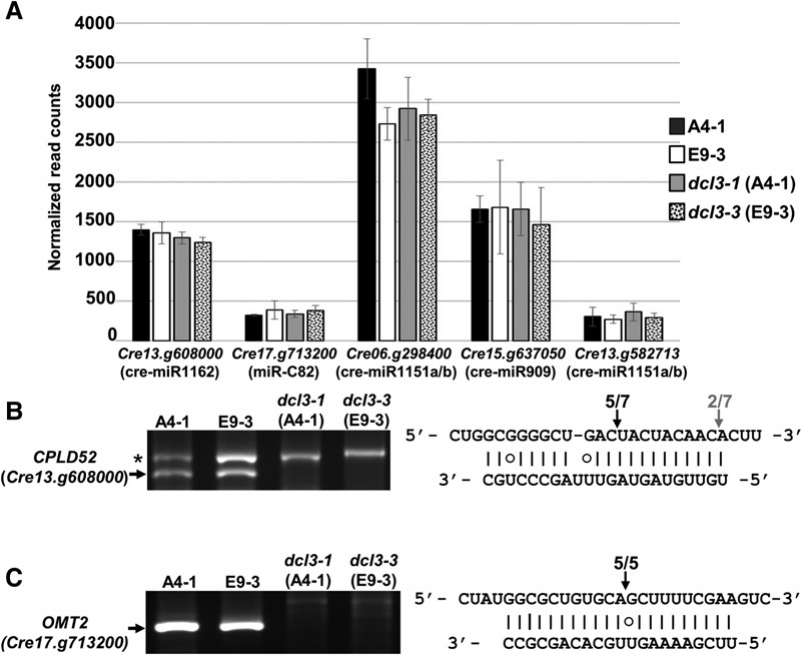
The effect of miRNA on mRNA accumulation. (*A*) Steady-state accumulation levels of previously reported miRNA targets (Molnár et al. 2007; Zhao et al. 2007) assessed as the number of normalized reads (*y*-axis) in RNA-seq data. Error bars for three independent samples are shown. The target genes with their corresponding miRNA are indicated. These miRNAs were predicted as either high confidence miRNAs (cre-miR1162, cre-miR1151a/b) or medium confidence miRNA (miR-C82) (see Table 1), with the exception of cre-miR909 that is a hairpin-derived siRNA also depleted in the *dcl3* mutant background. (*B*) 5′RACE to test the specific cleavage of *CPLD52* (*Cre13.g608000*) mediated by cre-miR1162. The asterisk indicates an unspecific PCR product. The PCR products were sequenced, and the *right* panel shows the 5′ terminus of these cleavage products aligned to the 5′ to 3′ mRNA sequence and the 3′ to 5′ miRNA. G:U base pairs are indicated by a circle. (*C*) 5′RACE to test the specific cleavage of *OMT2* (*Cre17.g713200*) mediated by the miR-C82 (Zhao et al. 2007) with the 5′ terminus of these cleavage products aligned to the 5′ to 3′ mRNA sequence and the 3′ to 5′ miRNA as in *B*.

The predicted and confirmed miRNA-targeted mRNAs from *C. reinhardtii* (Molnár et al. 2007; Zhao et al. 2007) were conspicuously absent from the list of differentially expressed RNAs (Supplemental Table S3). These RNAs were equally abundant in the RNA-seq data sets of wild-type and *dcl3* mutant lines (Fig. 5A) despite the presence of the miRNA guided mRNA cleavage products only in the RNA samples from the wild-type strains (Fig. 5B,C). Presumably the miRNA-directed cleavage products are present at only low abundance in these samples.

**Figure 6.**
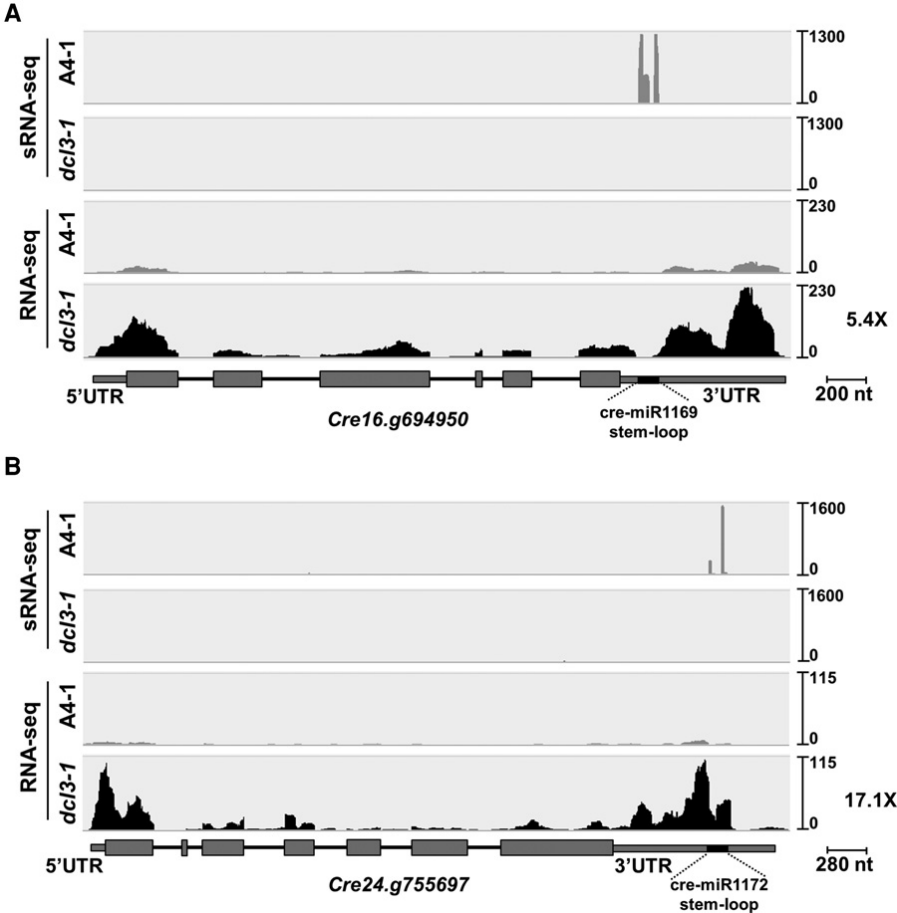
The effect of DCL3 on mRNAs with miRNA hairpin-like structures in the 3′ UTR. *Cre16.g694950* (serine/threonine kinase) (*A*) and *Cre24.g755697* (aminoglycoside 3′-phosphotransferase) (*B*) have the respective cre-miR1169 and cre-miR1172 precursors in their 3′ UTR. A schematic representation of both genes is shown with their exons (gray boxes) and introns (black solid lines) at the *bottom* of each panel. Light gray (A4-1 parental line) and black (*dcl3-1*) hills represent sRNA and mRNA read counts. Both panels show the results for one replicate of A4-1 parental line and its *dcl3-1* derivative knock out mutant. Overaccumulation (X-fold) of the indicated mRNA in *dcl3-1* regarding the A4-1 parental line is indicated. Two additional samples from this parental and mutant line combination and a biological triplicate from E9-3 parental and dcl3-3 derivative lines showed the same trend in both miRNA and mRNA accumulation (Table 1; Supplemental Table S3).

The primary effect of DCL3 on mRNA accumulation must be by direct cleavage of the mature mRNA as shown for two examples in Figure 6. These are mRNAs for which the exonic reads are more abundant in the *dcl3* mutant rather than wild-type samples (Fig. 6; Supplemental Table S3). The miRNA reads corresponding to the respective 3′ UTRs are conversely more abundant in wild-type samples (Fig. 6; Table 1). The other 45 mRNAs accumulating at higher level in the *dcl3* mutants correspondingly were from mRNAs containing miRNA/siRNA-like stem–loop structures in their coding or noncoding exons (Supplemental Table S3).

Most mRNAs with id-miRNAs, with the exception of the mRNA linked to cre-miR1154, were not affected by *dcl3* mutation (Supplemental Fig. S6). Based on these examples and results with the cre-miR1157 precursor (Fig. 4; Supplemental Fig. S5), we conclude that the DCL3 cleavage must be separate from mRNA splicing.

## Discussion

From our genetic analysis we have identified the DCL3 protein of *C. reinhardtii* as being responsible for sRNA biogenesis and mRNA accumulation. Our findings reinforce the idea that the miRNA silencing system in this alga is distinct from that of land plants and it may have features in common with the functional equivalent in animals. It is clear, however, from the phenotype of *dcl3* mutants that, unlike animals and land plants, miRNA silencing in *C. reinhardtii* is not required for normal growth and development. Our findings have implications for understanding the evolution and biological function of miRNA silencing in eukaryotes.

### miRNA silencing in *C. reinhardtii* is not typical of higher plants

DCL3 in *C. reinhardtii* has two features that are characteristic of similar proteins in nonplant organisms. The first of these is the absence of a PAZ domain (Supplemental Fig. S3) as with Dicer from the human parasite *Toxoplasma gondii*. This protozoan protein, together with the three DCLs from *C. reinhardtii*, forms a clade that is independent of both higher plant and animal DCLs (Fig. 1B in Braun et al. 2010).

The PAZ domain mediates the cleavage site selection in the miRNA precursor and size specification of the miRNA (MacRae et al. 2007) and, in its absence, it is likely that other proteins carry out these functions. Perhaps the large domain replacing PAZ in *C. reinhardtii* DCL3 is the anchoring site for such accessory functions in miRNA biogenesis. The proteins encoded by uncharacterized group II mutant loci are candidates for these accessory factors (Supplemental Table S1).

The second nonplant feature of DCL3 is a proline-rich domain on the amino terminal side of the two RNase III domains. There is a similar domain in an equivalent position in Drosha, the animal miRNA processor, which has an RNase III and lacks a PAZ domain (Ha and Kim 2014). These similarities prompt the hypothesis that *C. reinhardtii* DCL3 is both a Dcr and a Drosha with roles at several stages in miRNA biogenesis. The higher plant DCL1 is similarly involved in miRNA processing at early stages in addition to the final pre-miRNA cleavage (Brodersen and Voinnet 2009) but, unlike *C. reinhardtii* DCL3, it does not have any specific Drosha feature.

The miRNA genes of *C. reinhardtii,* like the DCL3 protein, also have nonplant characteristics. The most striking of these features is their overlap with protein-coding genes (Table 1). This is a frequent feature of animal miRNAs whereas higher plant miRNAs are, with only few exceptions, from noncoding RNA precursors. It is estimated in animals that ∼40% of the entire miRNA population are from introns (Kim et al. 2009) whereas, in plants, there are only three experimentally validated id-miRNAs (one and two in *Arabidopsis thaliana* and *Oryza sativa,* respectively) (Rajagopalan et al. 2006; Zhu et al. 2008; Joshi et al. 2012). In one of these examples the *Arabidopsis* DCL1 strongly represses *DCL1* mRNA abundance by cleavage of an miRNA precursor in intron 14 (Xie et al. 2003; Rajagopalan et al. 2006). In contrast, in *Chlamydomonas*, the id-miRNAs do not affect the abundance of the corresponding mRNA (Supplemental Table S3) or the miRNA-induced phenotype (Fig. 4) and so, even when higher plants have some id-miRNAs, there are major differences from *Chlamydomonas* miRNA features and mechanisms.

A second nonplant feature associated with the miRNA-related mechanisms of *C. reinhardtii* is with the UTR miRNAs. The mRNAs with miRNA structures in the UTR overaccumulated in the *dcl3* mutants, indicating that they are targeted for degradation by DCL3 in wild-type cells (Supplemental Table S3). An equivalent mechanism occurs with the mammalian *FSTL1* mRNA that is destabilized by Drosha during hs-miR198 biogenesis (Sundaram et al. 2013). Similarly the *DGCR8* mRNA is destabilized by Drosha cleavage via cleavage of a hairpin-like structure at the 3′ UTR, although there is no miRNA produced (Han et al. 2009).

There are 13 mRNAs in *C. reinhardtii* with miRNA in their UTRs (Table 1) of which eight accumulate at higher level in *dcl3* mutants (Supplemental Table S3). In addition, there are 26 mRNAs with siRNA precursors in their UTRs (Supplemental Table S3) and nine mRNAs with hairpin structures without associated sRNAs that are up-regulated in *dcl3* (Supplemental Table S3). It is likely, therefore, that there are at least 43 mRNAs in *C. reinhardtii* that may be subject to direct cleavage by DCL3. Remarkably, six of 13 UTR miRNAs bind AGO3 (Voshall et al. 2015), one of the three AGO proteins in *C. reinhardtii*. These observations prompt us to suggest that *C. reinhardtii* DCL3, like animal Drosha, has a dual role in mRNA regulation: It is first a ribonuclease that controls the levels of certain mRNAs by direct cleavage; and second, it is involved in biogenesis of sRNAs that act in *trans* to influence either mRNA accumulation or translation (Ma et al. 2013; Yamasaki et al. 2013; Voshall et al. 2015).

Finally, a third nonplant feature associated with *C. reinhardtii* miRNAs concerns the complementarity requirement for miRNAs to produce an effective down-regulation of their targets. Effective miRNA silencing in higher plants depends on near complete complementarity of the miRNA and its target (Liu et al. 2014) whereas, in *C. reinhardtii*, pairing in the miRNA seed region is sufficient to induce down-regulation (Yamasaki et al. 2013).

### Evolution of miRNA silencing in *C. reinhardtii*

Animal and plant miRNA pathways are very different and it is likely that they evolved separately from an ancestral RNA silencing pathway with Dicer proteins and small RNAs with 5′ phosphate and 3′ hydroxyl groups that bind to AGO proteins (Ghildiyal and Zamore 2009; Axtell et al. 2011). The algal/*Chlamydomonas* miRNA pathway is also distinct from that of higher plants, as discussed above, and we can envision either of two evolutionary scenarios to explain those differences. The first of these is that animal, higher plant, and algal miRNA pathways all evolved independently of each other. A second scenario is that an animal-like miRNA pathway evolved early and persisted in lower plant lineages, including the green algae and *C. reinhardtii*, although it was not retained in higher plants.

Our data are consistent with the second scenario because *C. reinhardtii* and animal miRNA pathways share the presence of a Drosha-like structure (absence of PAZ and presence of P-rich domain) of the miRNA processing enzyme (Supplemental Fig. S3), Drosha-like dual function exerted by the miRNA processing enzyme (Fig. 6; Supplemental Table S3), and miRNA association with introns or exons of RNA coding sequences (Table 1). In addition, as mentioned above, the animal and *C. reinhardtii* miRNA systems depend only on seed region complementarity (Yamasaki et al. 2013), and they both use VIG and TSN1 (Voshall et al. 2015; Ibrahim 2009).

At present there are insufficient data to resolve these two alternative scenarios, although the further characterization of additional class I –IV mutants (Supplemental Table S1) may shed more light on the evolutionary origin of miRNAs in *C. reinhardtii*.

### The role of sRNAs in *C. reinhardtii*

To explain the absence of physiological phenotype in our *dcl3* mutants in normal laboratory conditions as described here and in a description of another unrelated RNA silencing mutant of *Chlamydomonas* (Voshall et al. 2015) we propose that DCL3 has a role at certain stages of the life cycle or under conditions that have not yet been tested. A role under starvation of sulphate and/or phosphate is possible because these conditions affect sRNA profiles in *Chlamydomonas* (Shu and Hu 2012; Zheng et al. 2015). The DCL3-dependent silencing might also act redundantly with other silencing systems as indicated by the loss of transposon silencing in *C. reinhardtii* that was dependent on loss of function at both DCL1 and of a histone methyltransferase (Casas-Mollano et al. 2008). The availability of DCL3 mutants will now allow us to test these possibilities. We cannot, however, rule out the possibility that at least some of the *C. reinhardtii* sRNAs have a silencing-independent role.

## Methods

### Strain, culture conditions, and transformation

The *C. reinhardtii* cell-wall deficient strain CC-1883 (*cw15, NIA, NIT2, mt*^−^) was used in this study as wild-type background. It was obtained from the *Chlamydomonas* Resource Center (University of Minnesota) and grown in either solid or liquid 2-amino-2-(hydroxymethyl)-1,3-propanediol (TRIS)-acetate-phosphate (TAP) media (Harris 2009) at 25°C under continuous illumination. When indicated, cells were grown in nitrate TAP (TAP medium in which ammonium was replaced by the equivalent amount of nitrate).

For transformation, the indicated DNA cassettes were excised from their backbones, and ∼100 ng purified fragments were used for each transformation experiment. Transformations of mid-log-phase cells were done by electroporation following a previously described method (Shimogawara et al. 1998) in a Gene Pulser Xcell apparatus (Bio-Rad) with exponential electric pulses (2250 kV/cm, 10 µF). After recovery, cells were plated on solid media in the presence of starch.

### DNA oligonucleotides

DNA sequence of primers used in this study are listed in Supplemental Table S4.

### Plasmids

The nitrate-inducible amiRNA construct (ni-amiRNA) was generated from pMS539 (Schmollinger et al. 2010) by subcloning a *Xba*I/*Dra*I excised fragment that contains the Nit1 promoter/5′ UTR, amiRNA precursor, and terminator into *Xba*I/*Sma*I digested pSI103-1 (a derivative of pSI103) (Sizova et al. 2001). Unlike the original pMS539, the resulting plasmid confers resistance to paromomycin once integrated into the *C. reinhardtii* genome.

The amiRNA that targets *PSY* (*Cre02.g095092*) mRNA was designed using the Web MicroRNA Designer (http://wmd3.weigelworld.org/). The 21-nt amiRNA 5′-UGAUUUUGGAAGCGUUCGGCC-3′ was introduced in ni-amiRNA as a 90-nt double- stranded DNA (obtained by in vitro annealing of amiFor-PSY and amiRev-PSY primers) in its unique *Spe*I restriction site, following a previously described method (Molnár et al. 2009), to generate the ni-amiRNA-PSY.

The intron-derived cre-miR1157 precursor that lacks the cre-miR1157 stem–loop was amplified by PCR from ni-amiRNA with primers miR1157-Prec-For and miR1157-Prec-Rev, which carry tails in order to reconstitute the whole intron 22 from *Cre12.g537671* and add *Pml*I and *Pvu*II restriction sites at the 5′ and 3′ ends of the PCR product, respectively. This PCR product was cloned into pGEM-T Easy (Promega) to create pGEMT-miR1157. The gene splicing via overlap extension method (Horton et al. 1989) was used to generate the *Spect/intron*, a plasmid based on pALM32 (Meslet-Cladière and Vallon 2011) that carries the whole intron-derived cre-miR1157 (without the stem–loop precursor) in the middle of the *aadA* gene. A mix of three different DNA fragments was used as template for the overlapping PCR: (1) a PCR product obtained by amplification from pALM32 using the primers RBSC2_Pro-For and Spect+intron1157-Rev; (2) a PCR product obtained by amplification from pALM32 using the primers Spect+intron1157-For and RBSC2_3′UTR-Rev; and (3) a *Pml*I/*Pvu*II digested fragment from pGEMT-miR1157. The resulting PCR fragment was digested with *Kpn*I and cloned into *Sma*I/*Kpn*I digested pALM32 to generate the *Spect/intron* plasmid. *Spect/intron(mi)* was generated by cloning an amiRNA (5′-UAUGUACACAAUGCACUUCAG-3′), which targets the tryptophan synthase beta-subunit mRNA, into the *Spect/intron* plasmid by following the procedure described above. Site-directed mutagenesis of the splicing donor site in *Spect/intron(mi)* was carried out by two PCR steps, as previously described (Herlitze and Koenen 1990). The first round PCRs were done by using *Spect/intron(mi)* as template plus primer pair HSP70-For and Intron_Donor-Mut-Rev primers, or primers Intron_Donor-Mut- For and SpeI-Rev. These two PCR products were then used as template for the second round PCR with primers HSP70-For and SpeI-Rev. The resulting PCR product was digested with *Aat*II/*Spe*I and cloned by triple ligation with *Aat*II/*Hind*III and *Spe*I/*Hind*III digested fragments from *Spect/intron(mi)* to generate *Spect/Δintron(mi)*.

BAC clones 29A6 (A6) and 29M20 (M20) carrying the *C. reinhardtii* DCL3 genomic sequence were identified in a BAC library generated by Paul Lefebvre (University of Minnesota) by using the JGI v4 browser (http://genome.jgi-psf.org/Chlre4/Chlre4.info.html). The whole library, named CRCCBa, was obtained from the Clemson University Genomics Institute, and the indicated clones were isolated from *E. coli* glycerol stocks by using the QIAGEN Large-Construct Kit (Qiagen) following the manufacturer's instructions.

Plasmids pSI103-1 (J Moy, M LaVoie, C Silflow, unpubl.), pHyg3 (Berthold et al. 2002), and pALM32 (Meslet-Cladière and Vallon 2011) were obtained from the *Chlamydomonas* Resource Center (University of Minnesota).

### Mutant screen and mapping of mutagen integration sites

Two independent transgenic lines (here called A4-1 and E9-3 parental lines) carrying a functional ni-amiRNA cassette were grown on liquid TAP until mid-log-phase (this medium carries ammonium as the only nitrogen source, which represses the Nit1 promoter). Random insertional mutants were obtained by transformation of A4-1 and E9-3 with the corresponding resistance cassettes from pALM32 and pHyg3, respectively, following the transformation protocol described above. Mutant lines potentially affected in the miRNA silencing pathway were observed at 5–7 d after plating the cells in solid amiRNA induction media (TAP medium in which the ammonium was replaced for an equivalent amount of nitrate) at high light intensity (200 µmol photons m^−2^sec^−1^). Insertions were mapped in the *C. reinhardtii* genome by RESDA-PCR as previously described (González-Ballester et al. 2005). Primer pairs annealing across the insertion sites were used for easy genotyping of *dcl3* mutant lines by direct PCR, from a tiny amount of cells, by using the Phire Plant Direct PCR Kit (Thermo Scientific) according to the manufacturer's instructions.

### RNA extraction and analyses

RNA isolation and small RNA detection by Northern blot were carried out as previously described (Molnár et al. 2007). A detailed protocol can be found at http://www.plantsci.cam.ac.uk/research/davidbaulcombe/methods/downloads/smallrna.pdf/view. DNA primers corresponding to the reverse complementary sequence of the indicated miRNAs (amiRPSY-det, miR1151b-det, miR1157-det, miR1162-det, siRgypsy-det, miR_Cre07.g341100-det, miR_ Cre07.g344260-det, miR_Cre14.g623850-det, and miR_Cre16.g670000-det) (Supplemental Table S4) were radiolabeled with γ^32^P-ATP by the action of polynucleotide kinase and used to probe membranes with immobilized RNA samples. Radioactive signals were further detected with a phosphorimager.

The accumulation level of *PSY* mRNA was estimated by qRT-PCR from 5 µg DNA-free RNA. Briefly, RT was primed with random hexamers and SuperScript III (Invitrogen) following the manufacturer's guidelines. The PCR amplification step was carried out with primers PSY-qPCR-For and PSY-qPCR-Rev in the presence of the dsDNA-specific dye SYBR Green (Sigma) and monitored with a Chromo4 qPCR machine (Bio-Rad). The *RACK1* gene (*Cre06.g278222*) was used as an internal control for normalization. The delta-delta Ct method was used to calculate the differences in mRNA abundance.

RT-PCR was used to confirm splicing of the artificially generated, intron-derived, miRNA precursor. To do that, RT reaction was carried out as described above, whereas a normal PCR amplification step with primers Spect-For and Spect-Rev, which flank both sides of the intron, was done using the RT reaction as template.

5′ RNA ligase-mediated RACE was done as described (Llave et al. 2002) with the GeneRacer kit (Invitrogen). First PCR round was done with distal primers (PSY-Rev, OMT2-Rev and CPLD52-Rev), while nested primers were used for the second round PCRs (PSY_nested-Rev, OMT2_nested-Rev, CPLD52_nested-Rev). The final PCR fragments were gel purified using MinElute gel extraction kit (Qiagen) and cloned into pCRII vector (Invitrogen). Positive clones were further analyzed by DNA sequencing to map exact miRNA cleavage sites.

### Preparation of RNA libraries

Prior to preparing the sRNA libraries, samples carrying 10 µg total RNA were subjected to the FDF-PAGE method as previously described (Harris et al. 2015). The sRNA libraries were further prepared according to the TruSeq small RNA cloning protocol (Illumina) and run in an Illumina HiSeq 2000 (BGI HongKong).

Libraries for RNA-seq were prepared from poly(A) RNAs, which were purified from 50 µg total RNA by using the MicroPoly(A)Purist Kit (Ambion), following the manufacturer's instructions. Poly(A) RNA was used as starting material for the ScriptSeq v2 RNA-seq Library Preparation Kit (Illumina). Libraries were prepared following the manufacturer's protocol and run in an Illumina HiSeq 2000 (BGI HongKong).

### Analysis of sRNA high-throughput sequencing data

Illumina sRNA libraries were preprocessed using the ADDAPTS pipeline and tracking system (http://www.plantsci.cam.ac.uk/bioinformatics/addapts). After 3′ adaptor removal, all sequences <15 nt in length are discarded, and the remaining sequences are aligned against *C. reinhardtii* genome v5.0 using PatMaN (Prüfer et al. 2008). Only sequences with at least one perfect match are included. The initial sequencing data for each library and the number of reads obtained after each step are indicated in Supplemental Table S5. For the definition of sRNA producing loci, segmentSeq_2.2.1 (Hardcastle et al. 2012), available as part of Bioconductor, was used. This package takes the density of matches of sRNAs to the genome to determine regions corresponding to sRNA producing transcripts, taking into account replicate data. Segments with a higher than 0.9 posterior probability of being loci were used. Loci were subjected to differential expression analysis using baySeq 2.2.0 (Hardcastle and Kelly 2010). This package uses the negative binomial distribution for the count data produced by high-throughput sequencing and estimates its parameters using empirical Bayes, with the number of iterations determined by the parameter “sample size.” Models for different patterns of differential expression (including no differential expression) among the samples are specified, and the model with the highest posterior probability is used. The library scaling factor (surrogates for library size) has to be specified for each sample, and they were calculated by using the previously described quantile normalization (Bullard et al. 2010). This method sums all counts in each sample for which the value below the *q*th quantile of nonzero counts for that particular sample. Only those loci with a likelihood ≥0.9 of being differentially expressed in the specified model were considered.

A Python (v2.7.9) script was developed to count the number of overlaps between genomic annotations (Phytozome v5.5), repeat masker annotations (Phytozome v10.3), inverted repeats, tandem repeats, and miRNA precursors (these three last features were predicted as explained below) with the sets of differentially and nondifferentially expressed loci. Inverted repeats and tandem repeats were predicted by Inverted Repeat Finder v3.0.7 and Tandem Repeat Finder v4.0.7b, respectively (Benson 1999; Warburton et al. 2004).

### MiRNA prediction

The identification of miRNA precursors was performed by a multistep process, which first uses a combination of three different miRNA prediction algorithms: miRDP, miRDeep2 (with minimum score of 5), and miRCat (Mackowiak 2011; Yang and Li 2011; Stocks et al. 2012). These results were then combined to remove duplicate predictions, precursors with mature miRNAs with sizes outside 20–22 nt, and/or precursors with less than 100 sRNA reads. Finally, the last automated step was performed by removing those predicted miRNA precursors that did not overlap with those sRNA loci that had been previously identified by segmentSeq (see above). The resulting precursors were manually curated for the presence of miRNA* and defined miRNA stacks in an attempt to follow the standards of high confidence recommended by miRBase (Kozomara and Griffiths-Jones 2014). The number of identified miRNA precursors at each stage of the multistep process is shown in Supplemental Table S6. Detailed information about predicted miRNA precursors (exact location in the genome and nucleotide sequences of the corresponding mature miRNAs and miRNAs*) is shown in Supplemental Table S7.

### Analysis of RNA-seq high-throughput sequencing data

RNA-seq libraries were first analyzed with FastQC v0.11.2 for quality control (http://www.bioinformatics.babraham.ac.uk/projects/fastqc/). Trimmomatic 0.32 (Bolger et al. 2014) was then used for adaptor removal and trimming of bases with a quality score lower than 20. Reads shorter than 40 nt were discarded, and the remaining reads were subsequently aligned to rRNA, ncRNA, cpDNA, and mtDNA from *C. reinhardtii* using Bowtie 2 (Langmead and Salzberg 2012). Positive matches were discarded. Finally, the remaining reads were aligned with Bowtie 2 against the *C. reinhardtii* transcriptome (Phytozome v5.5). The initial sequencing data for each library and the number of reads obtained after each filtering step are indicated in Supplemental Table S5. Quantification of transcript abundance was performed using express 1.5.1 (Roberts and Pachter 2013). The est_counts and eff_length from express were then passed as input to baySeq (Hardcastle and Kelly 2010) for the differential expression analysis. Transcripts for which a likelihood ≥0.9 in the specified model were considered as differentially expressed.

## Data access

Small RNA-seq and RNA-seq data sets generated during this study have been submitted to the ArrayExpress database (EMBL-EBI; https://www.ebi.ac.uk/arrayexpress/) under accession numbers E-MTAB-3851 and E-MTAB-3852, respectively. Plasmids and strains generated in this study are available at the *Chlamydomonas* Resource Center (University of Minnesota).

## Supplementary Material

Supplemental Material
