# Supplementary material for: Most microRNAs in the single-cell alga Chlamydomonas reinhardtii are produced by Dicer-like 3-mediated cleavage of introns and untranslated regions of coding RNAs
Source: Genome Res. 2016 Apr;26(4):519–29. doi: 10.1101/gr.199703.115 (PMC4817775; doi:10.1101/gr.199703.115)
Supplement: Supplemental Material [file supp_26_4_519__index.html]

Most microRNAs in the single-cell alga Chlamydomonas reinhardtii are produced by Dicer-like 3-mediated cleavage of introns and untranslated regions of coding RNAs — Most microRNAs in the single-cell alga Chlamydomonas reinhardtii are produced by Dicer-like 3-mediated cleavage of introns and untranslated regions of coding RNAs — Supplemental Material 

# Most microRNAs in the single-cell alga *Chlamydomonas reinhardtii* are produced by Dicer-like 3-mediated cleavage of introns and untranslated regions of coding RNAs

## Supplemental Material

**Files in this Data Supplement:**

- Supplemental Figures and Legends.pdf
- Table S1.docx
- Table S2.xlsx
- Table S3.xlsx
- Table S4.xlsx
- Table S5.xlsx
- Table S6.xlsx
- Table S7.xlsx
