# Supplementary material for: Most microRNAs in the single-cell alga Chlamydomonas reinhardtii are produced by Dicer-like 3-mediated cleavage of introns and untranslated regions of coding RNAs
Source: Genome Res. 2016 Apr;26(4):519–29. doi: 10.1101/gr.199703.115 (PMC4817775; doi:10.1101/gr.199703.115)
Supplement: Supplemental Material [file supp_gr.199703.115_Supplemental_Figures_with_Legends.pdf]

SUPPLEMENTARY FIGURE LEGENDS

Supplementary Figure S1. Validation of the miRNA reporter system. (A) Detection by northern blot of the artificial (a)miRNA against *PSY* in total RNA samples from transgenic lines carrying the indicated constructs. These lines were grown in normal TAP until mid-log phase and then further incubated with nitrate TAP in the dark for 48 hours. (B) Phenotype in both normal and high light intensity conditions of transgenic lines analyzed in (A) when grown on solid media containing either ammonium or nitrate as the sole nitrogen source. (C) Relative levels of *PSY* mRNA in lines tested in (A) assessed by quantitative RT-PCR. (D) 5'RACE to test the specific cleavage of the *PSY* mRNA mediated by the amiRNA. Arrows and numbers indicate the positions and the frequencies of 5' termini of RACE products (top) aligned with the amiRNA (bottom). A schematic representation of the *PSY* mRNA with the location of the amiRNA cleavage site is also displayed.

Supplementary Figure S2. Effect of *dcl3* on expression of *PSY* mRNA. (A) Genotype by PCR of three independent alleles of *dcl3*. Top panel: schematic representation of *Cre07.g345900* (*DCL3*) indicating the primer pairs used for genotyping. Bottom panel: Analysis of PCR products from DNA triplicates of the indicated lines using the indicated primer pairs. (B) Relative levels of *PSY* mRNA in the indicated lines assessed by quantitative RT-PCR. The genetic background of each mutant strain is shown in parentheses. (C) Analysis of 5'RACE products produced by specific amiRNA-mediated cleavage of the *PSY* mRNA.

Supplementary Figure S3. Domain structure of RNaseIII-containing proteins. (A) The domains in the three DCL proteins from *C. reinhardtii* are shown in different colours (DEAD/DExH: helicase domain; Hel_C: helicase domain; DUF: DUF283, putative dicer dimerization domain; RIII: RNaseIII domain; P-Rich: proline-rich domain) with the size of the protein indicated in number of amino acids (aa) on the right hand side. (B) Domains in the indicated DCLs (top panel) and Drosha proteins (bottom panel) using the colour code as in (A) (PAZ: Piwi, Argonaute, Zwille domain). Protein sizes in aa are also indicated. Cr: *C. reinhardtii*; At: *Arabidopsis thaliana*; Hs: *Homo sapiens*; Dm: *Drosophila melanogaster*.

Supplementary Figure S4. Detection of newly identified high confidence miRNAs by northern blot. RNA samples from the indicated *C. reinhardtii* strains were probed for the presence of the specified miRNAs (for more details about these miRNAs, see Table 1). Asterisks indicate that the miRNA overlaps with the annotated gene but in reverse orientation.

Supplementary Figure S5. Characterisation of an artificial id-miRNA. (A) Transgenic *C. reinhardtii* lines, transformed with the indicated plasmids, at 7 days after plating on solid media that contains spectinomycin. (B) Schematic representation of constructs carrying the cre-miR1157 intron precursor inserted in the middle of the spectinomycin resistance gene coding sequence (details as in Figure 4A). The region in which the PCR primers (black arrows) anneal is indicated. (C) Analysis of RT-PCR products generated from total RNA of three independent transgenic lines transformed with the indicated construct. Genomic DNA and RT negative controls were also used as template in the PCR reaction for each of the samples. RT-PCR products corresponding to *spect/intron(mi)* transgenic lines were run in a different agarose gel at the same time than the other samples; hence, a white line separate the two images.

Supplementary Figure S6. The effect of *dcl3* mutation on the accumulation level of mRNAs with id-miRNAs. Steady-state accumulation levels of the indicated mRNAs (see also Table 1 for more information about intron-derived miRNAs) is assessed as the number of normalized reads (Y-axis) in RNA-seq data. Error bars for 3 independent samples are shown.

Valli_FigS1

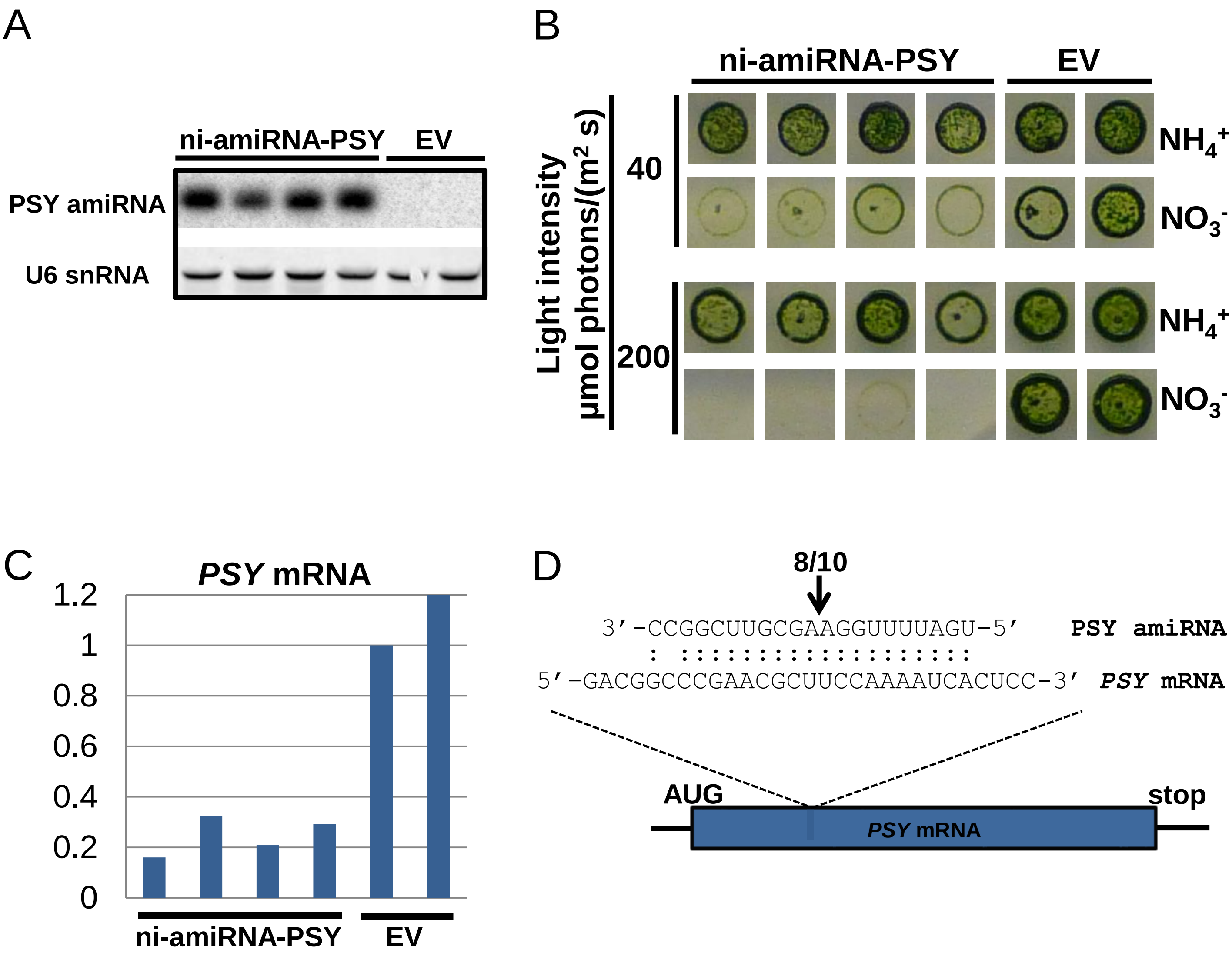
A
ni-amiRNA-PSY
EV
PSY amiRNA
U6 snRNA
B
ni-amiRNA-PSY
EV
Light intensity
μmol photons/(m2 s)
40
200
NH4+
NO3-
NH4+
NO3-
C
PSY mRNA
1.2
1
0.8
0.6
0.4
0.2
0
ni-amiRNA-PSY
EV
D
8/10
3′-CCGGCUUGCGAAGGUUUUAGU-5′
PSY amiRNA
5′-GACGGCCCGAACGCUUCCAAAAUCACUCC-3′
PSY mRNA
AUG
stop
PSY mRNA

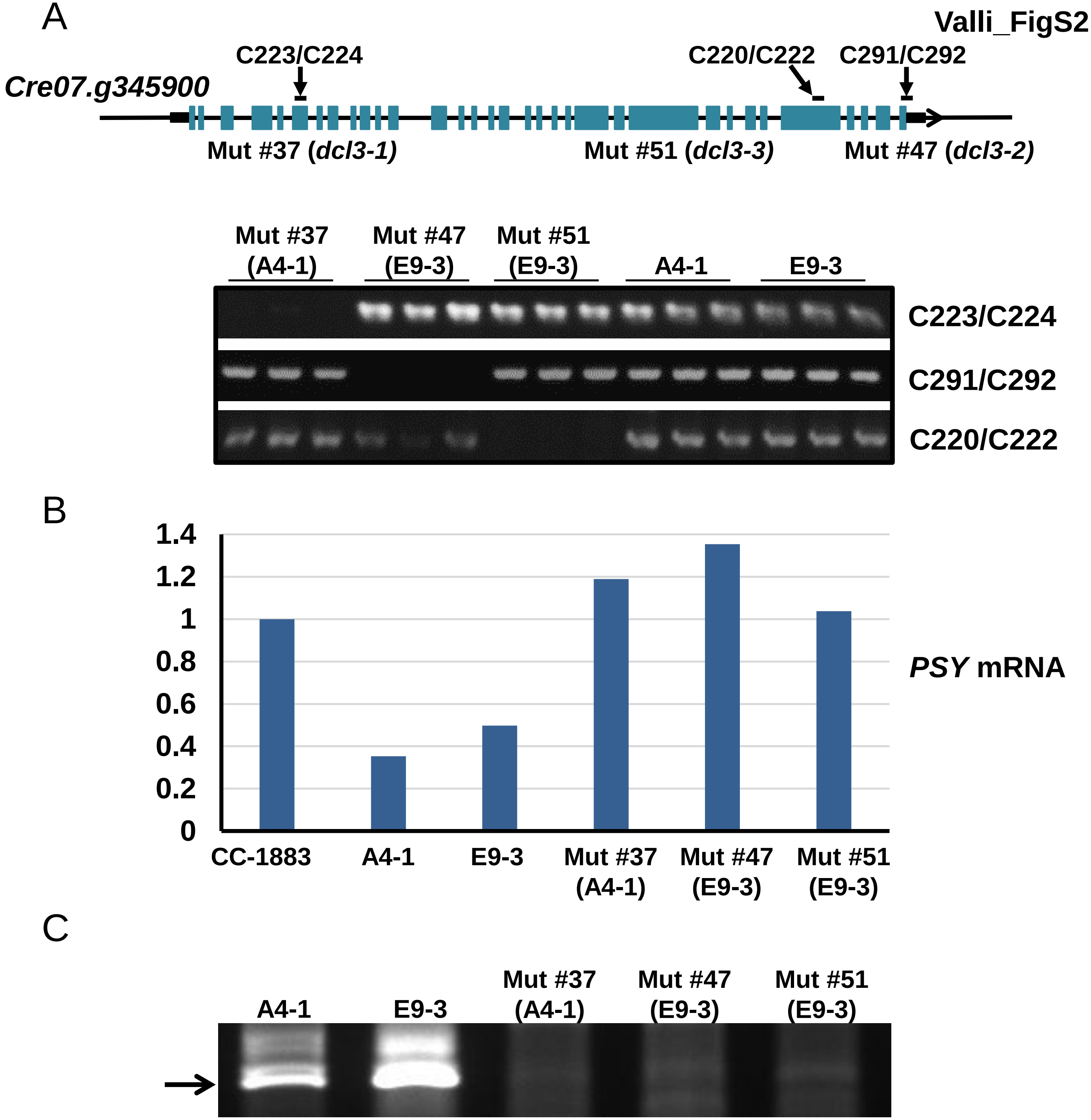
Valli_FigS2
A
C223/C224
C220/C222
C291/C292
Cre07.g345900
Mut #37 (dcl3-1)
Mut #51 (dcl3-3)
Mut #47 (dcl3-2)
Mut #37 (A4-1)
Mut #47 (E9-3)
Mut #51 (E9-3)
A4-1
E9-3
C223/C224
C291/C292
C220/C222
B
1.4
1.2
1
0.8
0.6
0.4
0.2
0
PSY mRNA
CC-1883
A4-1
E9-3
Mut #37 (A4-1)
Mut #47 (E9-3)
Mut #51 (E9-3)
C
A4-1
E9-3
Mut #37 (A4-1)
Mut #47 (E9-3)
Mut #51 (E9-3)

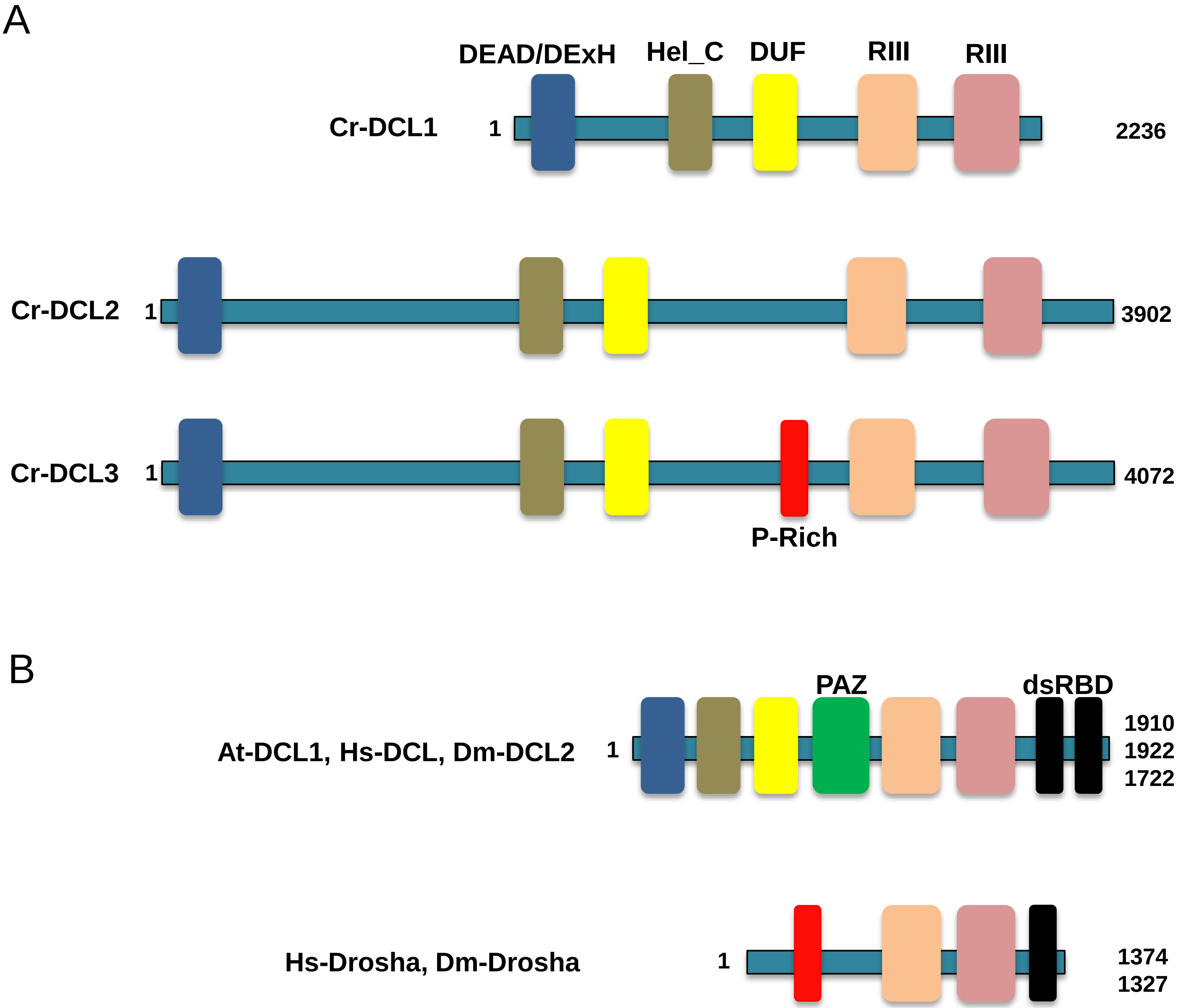
A
DEAD/DExH
Hel_C
DUF
RIII
RIII
Cr-DCL1
1
2236
Cr-DCL2
1
3902
Cr-DCL3
1
4072
P-Rich
B
PAZ
dsRBD
At-DCL1, Hs-DCL, Dm-DCL2
1
1910
1922
1722
Hs-Drosha, Dm-Drosha
1
1374
1327

Valli_FigS4

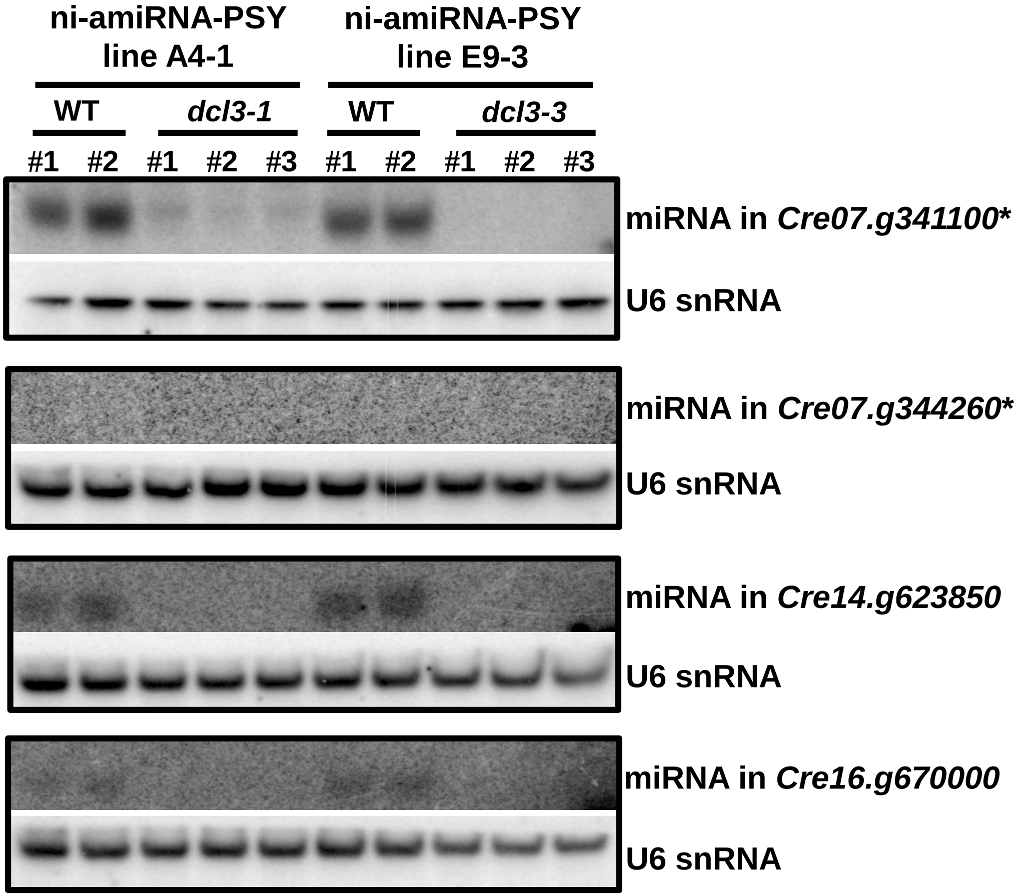
ni-amiRNA-PSY line A4-1
ni-amiRNA-PSY line E9-3
WT
dcl3-1
WT
dcl3-3
#1 #2 #1 #2 #3 #1 #2 #1 #2 #3
miRNA in Cre07.g341100*
U6 snRNA
miRNA in Cre07.g344260*
U6 snRNA
miRNA in Cre14.g623850
U6 snRNA
miRNA in Cre16.g670000
U6 snRNA

Valli_FigS5

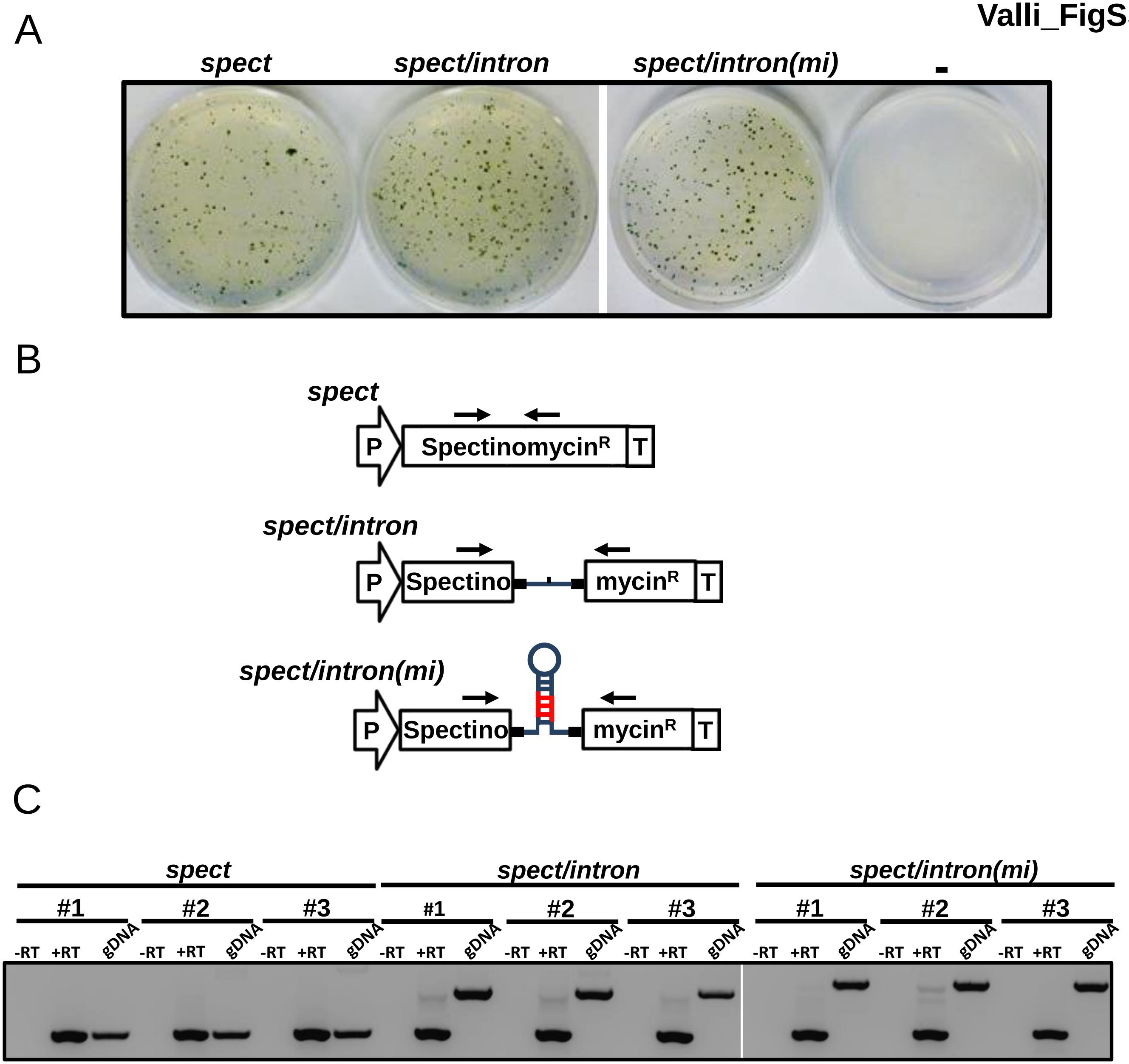

A
spect
spect/intron
spect/intron(mi)
-
B
spect
P
SpectinomycinR
T
spect/intron
P
Spectino
mycinR
T
spect/intron(mi)
P
Spectino
mycinR
T
C
spect
spect/intron
spect/intron(mi)
#1
#2
#3
#1
#2
#3
#1
#2
#3
-RT +RT gDNA
-RT +RT gDNA
-RT +RT gDNA
-RT +RT gDNA
-RT +RT gDNA
-RT +RT gDNA
-RT +RT gDNA
-RT +RT gDNA
-RT +RT gDNA

Valli_FigS6

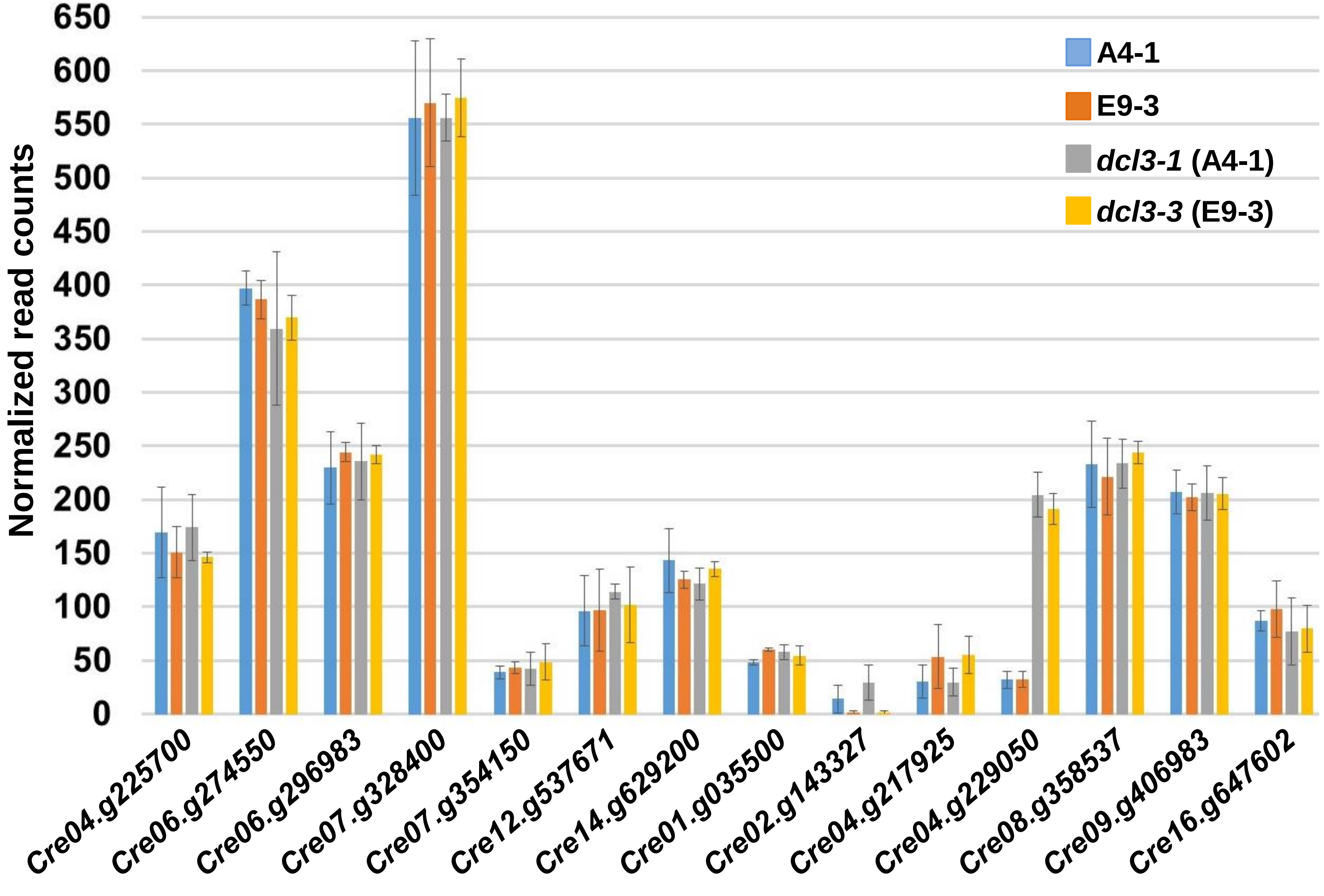

Normalized read counts
650
600
550
500
450
400
350
300
250
200
150
100
50
0
A4-1
E9-3
dcl3-1 (A4-1)
dcl3-3 (E9-3)
Cre04.g225700
Cre06.g274550
Cre06.g296983
Cre07.g328400
Cre07.g354150
Cre12.g537671
Cre14.g629200
Cre01.g035500
Cre02.g143327
Cre04.g217925
Cre04.g229050
Cre08.g358537
Cre09.g406983
Cre16.g647602
