# Supplementary material for: Most microRNAs in the single-cell alga Chlamydomonas reinhardtii are produced by Dicer-like 3-mediated cleavage of introns and untranslated regions of coding RNAs
Source: Genome Res. 2016 Apr;26(4):519–29. doi: 10.1101/gr.199703.115 (PMC4817775; doi:10.1101/gr.199703.115)
Supplement: Supplemental Material [file supp_gr.199703.115_Table_S1.docx]

Table S1: Diverse group of RNA silencing mutants that were isolated from the screen based on differences observed by the northern blot analysis.

| **Groups** | **I** | **II** | **III** | **IV** | **V** |
| --- | --- | --- | --- | --- | --- |
| Mutants | 11 | 6 | 1 | 4 | 1 |
| cre-miR1151b | +/- | - | -/+ | -/+ | -/+ |
| cre-miR1162 | -/+ | - | -/+ | +/- | +/- |
| PSY amiRNA | +/- | - | -/+ | - | -/+ |
| *gypsy* siRNA | + | - | -/+ | - | + |
| Diffused bands | - | - | + | - | - |
